# TREFEX: Trend Estimation and Change Detection in the Response of MOX Gas Sensors

**DOI:** 10.3390/s130607323

**Published:** 2013-06-04

**Authors:** Sepideh Pashami, Achim J. Lilienthal, Erik Schaffernicht, Marco Trincavelli

**Affiliations:** Centre for Applied Autonomous Sensor Systems, Örebro University, SE-70182 Örebro, Sweden; E-Mails: achim.lilienthal@oru.se (A.J.L.); erik.schaffernicht@oru.se (E.S.); marco.trincavelli@oru.se (M.T.)

**Keywords:** metal oxide sensors, open sampling system, change point detection, trend filtering

## Abstract

Many applications of metal oxide gas sensors can benefit from reliable algorithms to detect significant changes in the sensor response. Significant changes indicate a change in the emission modality of a distant gas source and occur due to a sudden change of concentration or exposure to a different compound. As a consequence of turbulent gas transport and the relatively slow response and recovery times of metal oxide sensors, their response in open sampling configuration exhibits strong fluctuations that interfere with the changes of interest. In this paper we introduce TREFEX, a novel change point detection algorithm, especially designed for metal oxide gas sensors in an open sampling system. TREFEX models the response of MOX sensors as a piecewise exponential signal and considers the junctions between consecutive exponentials as change points. We formulate non-linear trend filtering and change point detection as a parameter-free convex optimization problem for single sensors and sensor arrays. We evaluate the performance of the TREFEX algorithm experimentally for different metal oxide sensors and several gas emission profiles. A comparison with the previously proposed GLR method shows a clearly superior performance of the TREFEX algorithm both in detection performance and in estimating the change time.

## 1. Introduction

Detecting significant changes in the response of metal oxide (MOX) gas sensors is important for many applications such as gas leak detection in coal mines [1,2], large scale pollution monitoring [3,4] or the mapping of gas detection events [5]. A significant change (also frequently called an event) in the response of MOX sensors can indicate, e.g., the activity of a gas source or the sudden presence of specific compounds in a mixture of others. In the applications of interest, gas sensors are typically deployed in an OSS (open sampling system, i.e., directly exposed to the environment, without a sensing chamber and a sampling mechanism) since continuous monitoring is often crucial, and restrictions in costs and payload pose stringent limitations on the hardware that can be considered.

In this paper, we address the problem of detecting change points from the response of a MOX sensor or sensor array in OSS configuration. This problem is challenging because the sensor response deviates strongly from the “clean” signal that can be obtained in a controlled setting. The gas transport mechanisms in natural environments are dominated by turbulence and advection. This causes the sensor response to fluctuate continuously [6]. In addition, external factors such as humidity and temperature are also influencing the sensor readings [7]. Consequently, change point detection cannot be achieved by trivial threshold based methods.

The method introduced in this paper allows to interpret the response of MOX sensors in OSS configuration and to detect change points. This is important for a range of applications. Many locations of interest for monitoring of dangerous gases suffer from difficulties in wireless communication, e.g., in coal mines the radio signal is often unstable and of poor quality [1,2]. This poses problems in transferring lengthy data streams like time series obtained by MOX sensor readings collected with a wireless sensor network or mobile robots. Transferring small amounts of data representing only significant events rather than all sensor readings is more suitable for these scenarios. When gas sensors are mounted on a mobile robot, detection of change points in the signal is also important for detecting when the mobile robot enters or exits an odour plume or when the sensed chemical compound changes [8]. Finally, large scale pollution monitoring projects (such as CitiSense [3] and Air Quality Egg [4]) consider personal air-quality devices. Change point detection running locally on these devices can decrease energy consumption (continuous transfer of sensor readings is not necessary), and can help to address calibration issues [9].

In this paper we propose a change point detection algorithm explicitly designed for metal oxide (MOX) gas sensors. The aim of the proposed algorithm is to identify events such as the sudden exposure of the sensors to a gas, a sudden change of concentration of a gas or the change of gas to which the sensors are exposed. The detected change points can then be used as inputs by higher level estimation algorithm or decision systems. MOX gas sensors are the most common sensing technology for use in OSS because of their high sensitivity to a multitude of compounds, their simple operation and electronic interface, and their low price. MOX sensors are characterized by a slow response dynamics, and even slower recovery. Therefore, sudden changes in the exposure of a MOX sensor manifest as an exponential function in the sensor response. In this respect, a MOX sensor can be considered as a nonlinear asymmetric low pass filter, with different dynamics in the response and recovery phase. The TREnd Filtering with EXponentials (TREFEX) algorithm takes into account explicitly this characteristic behaviour of MOX sensors and tries to model the underlying trend of a sensor response as a piecewise exponential function. The proposed algorithm is very efficient from a computational viewpoint since it is formulated as a convex optimization problem. The TREFEX algorithm has one regularization parameter to be set by the user. In this article we also propose a method for setting the regularization parameter automatically, which requires few iterations of the algorithm. Finally, since MOX sensors are often deployed in arrays to achieve a level of selectivity that cannot be attained with a single sensor, we extend the proposed algorithm to be able to deal with a sensor array.

The rest of this paper is organized as follows. Section 2 presents related works in gas sensing with an OSS, change point detection in time series and trend filtering. The experimental setup with which the algorithms have been tested is described in Section 3, after which the proposed change point detection algorithm, the parameter selection strategy, and the extension to the sensor array configuration are explained in Section 4. Results are then presented in Section 5, first for the case of single sensor change point detection and then considering change point detection using multiple sensors. Finally, Section 6 concludes the paper and presents future work.

## 2. Related Works

Change detection in the activity of a gas source based on the response of an array of MOX gas sensors is related to the study of change point detection in the domain of the time series analysis. It has been studied for a wide range of applications such as quality control [10], climatology [11], image edge detection [12], and monitoring of land-cover changes [13]. The wide range of applications corresponds to very different solutions for the change point detection problem. Variations span from on-line to off-line algorithms and include multivariate or univariate approaches for detecting additive or multiplicative changes [14]. Keeping our area of applications in mind, we do not consider algorithms that require prior information about the position of the change point [15].

The first type of change detection solution is detecting deviations from normal behaviour based on a simple distance measure, e.g., for quality control applications [10] when the measurements fall out of a predefined range. A second category of algorithms analyse sequentially whether a change point exists by comparing the data before and after a hypothetical change point. These techniques often use statistical approaches both in a model-based and model-free fashion. Popular algorithms, inspired by frequentist statistics, include the Generalized likelihood Ratio (GLR) test [14], the Marginalized Likelihood Ratio (MLR) [16] and the CUmulative SUM (CUSUM) algorithm [14]. Instead of explicitly computing statistical parameters of the signal before and after an assumed change point, machine learning approaches such as one class Support Vector Machines were also suggested to detect change points [17]. Third, changes can be detected estimating the underlying trend of a signal as a piecewise function and then considering the connections between consecutive pieces as change points. Clearly these methods are model-based methods and rely on the assumption that the shape of the trend is known. Two examples are piecewise linear segmentation [18,19] and piecewise-constant segmentation [20]. These methods are usually off-line. The TREFEX algorithm proposed in this paper falls into the last category. Since the step response of MOX sensors can be approximated by an exponential function, the TREFEX algorithm segments a signal into a piecewise exponential trend.

## 3. MOX Sensors and Experimental Setup

In the following, we will discuss the characteristics of the sensors that we consider in this paper and present the setup of experiments used to obtain the data.

### 3.1. Metal Oxide (MOX) Gas Sensors

Metal oxide (MOX) gas sensors are, by far, the most widely used in electronic nose and mobile robotics olfaction applications. MOX gas sensors are conductometric sensors, where a change in the conductance of the oxide is measured when a gas interacts with the sensing surface. The change in conductance is approximately linearly proportional to the logarithm of the concentration of the gas over a range of concentrations [21]. There are two types of MOX sensors: *n*-type and *p*-type [22,23]. The response of a MOX sensor results from chemosorption and redox reactions at the surface. Since the rate of such reactions is dependent on the temperature and on the material of the surface, it is clear that the doping material of the sensing surface and the operating temperature considerably affect the sensor characteristics [21]. Typical temperatures for the sensing surface of MOX sensors lie between 300 °C and 500 °C.

**Figure 1.**
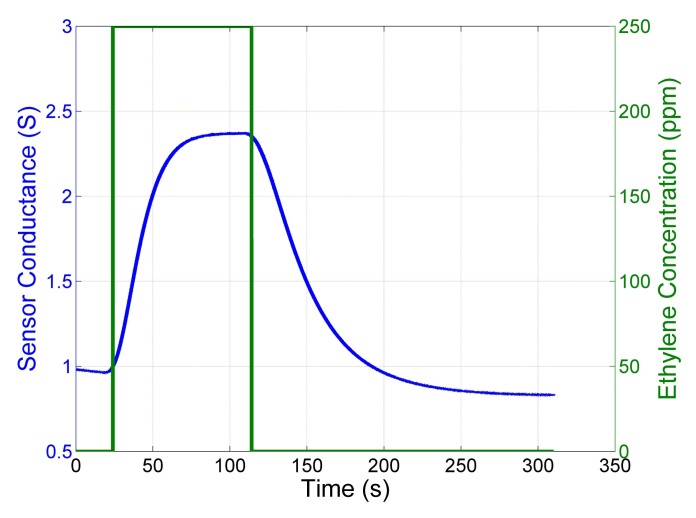
Response of a Figaro TGS2600 to a pulse of 250 ppm of ethylene obtained in a closed sampling system.

One of the main drawbacks of MOX sensors for open sampling applications is the slow response time and the even slower recovery time. Figure 1 shows the response time and recovery time in a closed sampling system without disturbances like turbulence and advection of the airflow. The MOX sensor was exposed to a pulse of ethylene and the sudden variation in the exposure of the sensor generates exponentials in the response. Those exponentials have different time constants depending on whether the conductance of the sensor increases (faster) or decreases (slower). This observation forms the foundation of the proposed change point detection algorithm, even if in an OSS additional disturbances affect the signal.

### 3.2. Experiments and Data Collection

**Figure 2.**
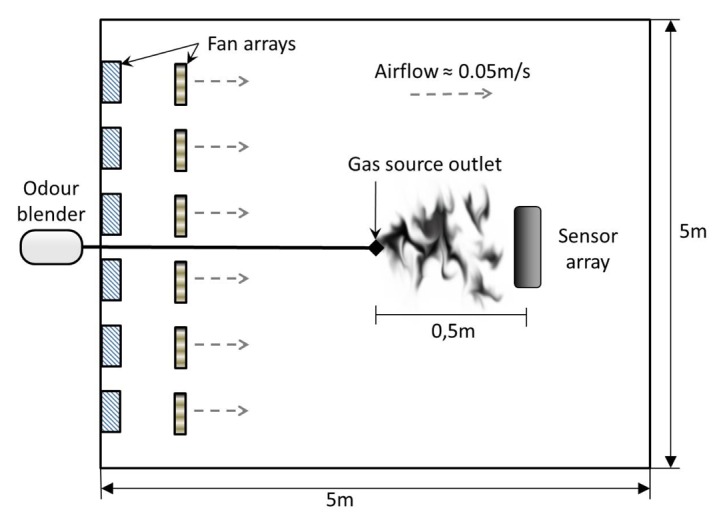
Schematics of the experimental room.

Experiments were carried out with static sensors in a 5 × 5 × 2 *m*^3^ closed room in which an artificial airflow of approximately 0.05 m/s was induced, see Figure 2. The airflow is created using two arrays of four fans, one placed on the floor and one on the wall. The gas source is an odour blender, a device developed by Nakamoto et al. [24], which allows fast switches in between different mixtures of compounds with a variable concentration. The outlet of the olfactory blender is placed on the floor 0.5 m upwind with respect to an array of 11 commercial metal oxide gas sensors from Figaro Engineering [25] (TGS2600 × 2, TGS2602, TGS2611, TGS2620) and e2v Technologies [26] (MiCS 5521 × 2, MiCS2610, MiCS2710, MiCS5121, MiCS5135). The selected sensors have overlapping sensitivity and they respond to a wide range of target compounds. The airflow at the outlet of the odour blender is set to 1 L/min. The sampling rate of the sensors is 4 Hz.

The two compounds selected for these experiments are ethanol and 2-propanol. Both ethanol (molecular weight 46 g/mol) and 2-propanol (molecular weight 60 g/mol) are heavier than air (average molecular weight 29 g/mol), and therefore will tend to create a plume at the ground level. The two substances have a similar saturated vapour pressure, namely 5.8 kPa for ethanol and 4.2 kPa for 2-propanol, which means that they have a similar tendency to evaporate. Moreover, the MOX gas sensors have a comparable sensitivity to the two substances. This is important in order to obtain similar sensor responses for both analytes, thus avoiding having to address a trivial instance of the change detection problem.

**Figure 3.**
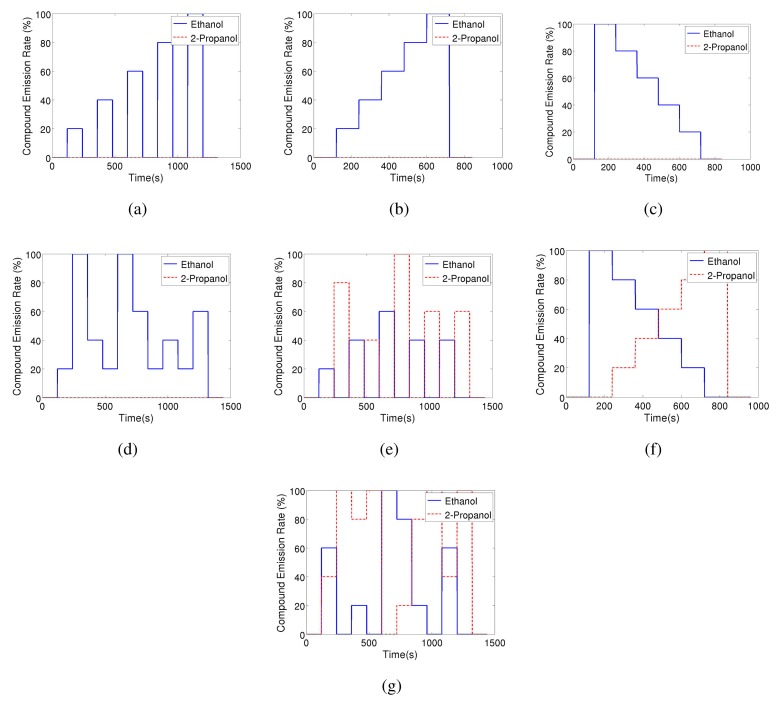
Gas source emission strategies. Strategies (**a**–**d**) are displayed only for ethanol (they are repeated identically also with 2-propanol as target gas). For the randomized strategies, *i.e.*, (**d**), (**e**), and (**g**) one exemplary instance is displayed. (a) Steps; (b) Ascending Stairway; (c) Descending Stairway; (d) Random Stairway; (e) Random Switching; (f) Mixture Stairway; (g) Random Mixture.

In order to create a database that allows studying the dynamic behaviour of the sensors when consecutively exposed to different analytes, seven different odour emitting profiles have been applied. For all these profiles the gas source emits clean air for two minutes and the signal of sensors during this period is assumed as a baseline. Also, at the end of all the experiments the source emits clean air for 2 min. Figure 3 shows the intensity profile for the gas source in the various emission strategies. A total of 54 experimental runs have been performed.

The control signal of the odour blender is used as ground truth for the change point time and provides the time at which the source changes the emission modality. However, in order to know the change point time at the sensors' location, we need to estimate the time it takes the gas to travel from the gas source to the sensor location. Since the sensors are placed 0.5 m away from the location of the source outlet and a steady air flow of 0.05 m/s is induced, the delay time between change times at source and sensor location is estimated to be 10 s. The estimation of the delay has been validated with a cross correlation analysis between the control signal of the odour blender and the signal of the MOX sensors in the Steps experiments (see Figure 3(a)).

## 4. Algorithm

The key idea behind the proposed change detection approach is that a change in the emission modality of a gas source appears as an exponential trend in the response of MOX sensors. MOX sensors, due to their long response and recovery times, can be modelled as a first order system whose step response is indeed an exponential [27]. The proposed method interprets the sensor response by fitting piecewise exponential functions with different time constants for the response and recovery phase. The number of exponentials is determined automatically using an approximate method based on *l*_1_-norm regularization. This asymmetric exponential trend filtering problem is formulated as a convex optimization problem, which is particularly advantageous from the computational point of view. Section 4.1 presents an introductory discussion on piecewise linear trend filtering based on [18], Section 4.2 presents the change point detection algorithm for a single sensor, Section 4.3 presents a strategy for selecting the parameter of the algorithm, and Section 4.4 extends the algorithm to the case where a sensor array is available.

### 4.1. Piecewise Linear Trend Filtering

In this Section we introduce the main ideas behind piecewise linear trend filtering proposed in [18] to prepare the discussion of the TREFEX algorithm presented in the following sections. The method falls in the framework of regularized regression and solves the following optimization problem

(1) $$\underset{\text{x}}{\text{minimize}}\;{\left\|\text{x}-\text{y}\right\|}_{2}^{2}+{\lambda \left\|\text{DDx}\right\|}_{1}$$

where ***y*** is the sensor response, ***x*** is the trend to be estimated, and ***D*** is the *N* × *N* matrix operator that calculates first-order differences:

$$\text{D}=\left[\begin{array}{ccccc} -1 & 1 & & & \\ & -1 & 1 & & \\ & & \ddots & \ddots & \\ & & & -1 & 1\\ & & & & -1 \end{array}\right]$$

**Figure 4.**
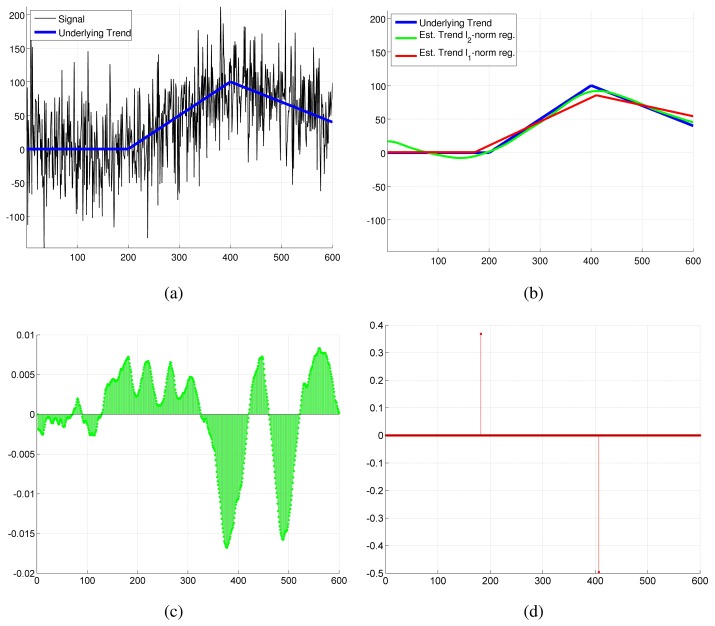
Toy example that illustrates the difference between *l*_1_-norm and *l*_2_-norm trend filtering. Sub-figure (**a**) illustrates a signal that is characterized by a piecewise linear trend with superimposed white Gaussian noise. Sub-figure (**b**) shows the true trend and the estimated trends using *l*_1_-norm and *l*_2_-norm regularization (the regularization parameter is set to *λ* = 50 in both cases). Sub-figures (**c**) and (**d**) shows the residuals of the regularization term ***DDx*** obtained respectively for the *l*_2_-norm and *l*_1_-norm. Notice that the scale of the ordinate axis is different for the plots in sub-figures (c) and (d). (a) Signal and underlying piecewise linear trend; (b) Trend and estimated *l*_1_-norm and *l*_2_-norm trends; (c) l_2_-norm residuals ***DDx***; (d) *l*_1_-norm residuals ***DDx***.

*λ* ≥ 0 is a regularization parameter used to control the trade-off between the deviation of the estimated trend from the signal ${\left\|\text{x}-\text{y}\right\|}_{2}^{2}$ and the smoothness of the trend encoded by ║***DDx***║**_1_**. For the case of piecewise linear filtering, smoothness is encoded as minimization of the second derivative, which for a line (assumed in linear trend filtering) is exactly equal to zero. The use of the *l*_1_-norm in the regularization term is the main difference between the trend filtering method proposed in [18] and the well known Hodrick-Prescott filtering [28] that uses instead an *l*_2_-norm regularizer. The *l*_1_-norm is used to induce sparsity in the smoothness term, *i.e.*, a solution for which the smoothness term is exactly zero for most of the points and greater than zero in few points. This results in a trend that is “mostly linear” with few sharp kink points, opposed to the trend found with a *l*_2_-norm regularizer that intuitively never “bends too much” but is also not a perfect line (the *l*_2_-norm does not induce sparsity). Figure 4 presents a toy problem that gives a practical example of this concept. Notice how the trend obtained with the *l*_1_-norm preserves the piecewise linear shape, with clear kink points between the lines, while the *l*_2_-norm trend is a smoothed-out version of the original trend where the break points between the line pieces are not clear. This is reflected in the residuals ║***DDx***║ that are equal to zero except at the kink points for the *l*_1_-norm regularization, while for the *l*_2_-norm regularization they are always very small but rarely exactly equal to zero. This makes *l*_1_-norm regularization more suited to detect change points, as the change points can be detected by the non-zero elements in the kinks vector.

### 4.2. Single Sensor

In this paper we propose to model, instead of a piecewise linear trend, a piecewise exponential trend for capturing the sensor response induced by abrupt changes in the emission of the gas source. This is motivated by the response characteristics of MOX sensors, outlined in Section 3.1. Exponential functions are characterized by the following relationship:

(2) $$\frac{d^{n-1}x}{dt^{n-1}}=-\tau \frac{d^{n}x}{dt^{n}}$$

where *τ* is the time constant of the exponential function. Since high order derivatives are noisy, we limit our attention to first and second derivatives and we write down the relationship for discrete time:

(3) Dx=−τDDx

where ***D*** is again the matrix operator that calculates first-order differences. Therefore, the optimization problem for detecting piecewise exponential trends can be defined in the following way:

(4) $$\underset{\text{x}}{\text{minimize}}{\;\left\|\text{x}-\text{y}\right\|}_{2}^{2}+\lambda {\left\|\;(\text{I}+\tau \text{D}\;)\text{Dx}\right\|}_{1}$$

The regularization term now encodes the tendency to fit an exponential by rewarding trends that comply with Equation (3). This formulation is however not suitable for MOX sensors since MOX sensors are slower in the recovery than in the response phase. Therefore it is needed to introduce exponentials with different time constants, namely *τ*_+_ and *τ*_−_, for the response and recovery phase. These constants have to be known or determined experimentally for each sensor. The optimization problem can be then modified in the following way:

(5) $$\begin{array}{cc} \underset{\text{x},{\text{d}}_{+},{\text{d}}_{-}}{\text{minimize}} & {\left\|\text{x}-\text{y}\right\|}_{2}^{2}+\lambda \;({\left\|\;(\text{I}+\tau_{+}\text{D}\;){\text{d}}_{+}\right\|}_{1}+{\left\|\;(\text{I}+\tau_{-}\text{D}\;){\text{d}}_{-}\right\|}_{1}\;)\\ \text{subject to} & {\text{d}}_{+}≽\text{Dx}\\ & {\text{d}}_{+}≽0\\ & {\text{d}}_{-}≼\text{Dx}\\ & {\text{d}}_{-}≼0\\ & {\text{d}}_{+}+{\text{d}}_{-}=\text{Dx} \end{array}$$

where *τ*_+_ and *τ*_−_ are the time constants of the response and decay phase respectively. The variables ***d***_+_ and ***d***_−_ and the corresponding linear inequality constraints were introduced to model the derivative of the trend for response and decay phases. Exploiting the equality constraint ***d***_+_ + ***d***_−_ = ***Dx*** the variable *d*_−_ can be eliminated, and therefore the problem can be written in the following, more compact, form:

(6) $$\begin{array}{cc} \underset{\text{x},{\text{d}}_{+}}{\text{minimize}} & {\left\|\text{x}-\text{y}\right\|}_{2}^{2}+\lambda \;({\left\|\;(\text{I}+\tau_{+}\text{D}\;){\text{d}}_{+}\right\|}_{1}+{\left\|\;(\text{I}+\tau_{-}\text{D}\;)\;(\text{Dx}-{\text{d}}_{+}\;)\right\|}_{1})\\ \text{subject to} & {\text{d}}_{+}≽\text{Dx}\\ & {\text{d}}_{+}≽0 \end{array}$$

The vector of kinks *k* between subsequent exponentials can be trivially calculated as the sum of the arguments of the two *l*_1_-norms in Equation (6):

(7) $$k=\left|\;(\tau_{+}-\tau_{-}\;)\text{D}{\text{d}}_{+}+(\text{I}+\tau_{-}\text{D}\;)\text{Dx}\right|$$

Change points are declared when *k* > 0.01. Once a change point has been declared, no more change points will be declared until ***Dx*** < 0.0001. This is to avoid that once the signal is already changing, multiple alarms would be triggered for slight imperfections in the estimated trends. These two values are arbitrarily chosen small numbers introduced to cope with numerical inaccuracies and with the fact that *l*_1_-norm is an approximation of the *l*_0_-norm (the number of non-zero elements in a vector) that would make the values of *k* exactly zero for samples that are not considered change points.

The optimization problem (6) is a convex optimization problem that can be easily reformulated as a Quadratic Program (QP). This problem can be solved with standard convex optimization methods that are implemented by optimization packages available on-line such as CVX [29] or Gurobi [30].

### 4.3. Parameter Selection

**Figure 5.**
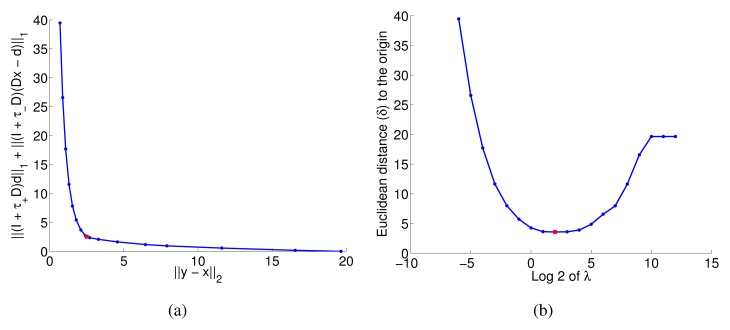
Trade-off curve for an experiment with the Random Stairway strategy, considering the response of the MiCS 2610 sensor for *λ* = [2^−6^,2^12^]. The selected value, *λ* = 2^2^, is highlighted with a red square.

The regularization parameter *λ* ≥ 0 is used to control the trade-off between how close the estimated trend reproduces the signal $\left({\left\|\text{x}-\text{y}\right\|}_{2}^{2}\right)$ and the smoothness of the signal encoded by the regularization term ${\left\|\;(\text{I}+\tau_{+}\text{D}\;){\text{d}}_{+}\right\|}_{1}+{\left\|\;(\text{I}+\tau_{-}\text{D}\;)\;(\text{Dx}-{\text{d}}_{+})\right\|}_{1}$. If *λ* = 0 the estimated trend *x* would be exactly equal to the signal *y*, while if *λ* → ∞ the estimated trend *x* would be the best fit (single) exponential to the signal *y*. Clearly, the appropriate values for *λ* lie between these two extrema. Figure 5(a) shows the curve obtained by plotting the two terms of the objective function for an experiment using the MiCS2610 sensor for various values of *λ*. An appropriate selection criterion for the parameter *λ* is to choose the *λ* for which the trade-off curve attains minimum Euclidean distance *δ* to the origin. Indeed, if for a value of *λ* the trade-off curve would pass through the origin, it would mean that there exists a perfectly smooth signal that passes through all the data points. As can be seen in Figure 5(b), the Euclidean distance shows a clear minimum and therefore it is an easy function to optimize.

Analogous to [18], we can show that there exists a *λ*_*MAX*_ at which the estimated trend becomes a single exponential. For values of *λ* beyond that point the solution does not change (this appears as a constant value for sufficiently large values of *λ* in Figure 5(b)). To derive *λ*_*MAX*_ we rewrite problem (6) as an equivalent QP and then make use of the KKT optimality conditions [31] in order to derive the following equation:

(8) $$\begin{array}{cc} \lambda_{\text{MAX}} & =\text{min}({\left(H_{+}^{T}H_{+}\right)}^{-1}H_{+}y,{\left(H_{-}^{T}H_{-}\right)}^{-1}H_{-}y)\\ H_{+} & =(I+\tau_{+}D)D\\ H_{-} & =(I+\tau_{-}D)D \end{array}$$

Given that we now know an interval [0, *λ*_*MAX*_] that contains the optimal value of lambda *λ*_∗_, we can use a standard bracket baseline search method like bisection or golden search [32] to minimize the distance of the trade-off curve from the origin.

In this work we choose the golden search method since it has the best worst case performance. This constitutes an efficient method for automatically selecting the only parameter of the algorithm, which implies the solution of few convex optimization problems. The proposed algorithm is then parameter free, which is a great advantage from the user point of view.

### 4.4. Sensor Array

Given their comparatively poor selectivity, MOX sensors are rarely deployed individually, but often they are included in a sensor array that can provide the desired selectivity as a whole. The proposed algorithm can be extended to be able to work on the response of a sensor array in the following way:

(9) $$\begin{array}{cc} \underset{\text{x},{\text{d}}_{+}}{\text{minimize}} & \sum_{t=1}^{N}{\left\|{\text{x}}_{t}-{\text{y}}_{t}\right\|}_{2}^{2}+\lambda \;(\sum_{t=3}^{N}{\left\|\;(\text{I}+\tau_{+}\text{D}\;){\text{d}}_{{+}_{t}}\right\|}_{p}+\sum_{t=3}^{N}{\left\|\;(\text{I}+\tau_{-}\text{D}\;)\;(\text{D}{\text{x}}_{t}-{\text{d}}_{{+}_{t}}\;)\right\|}_{p}\;)\\ \text{subject to} & {\text{d}}_{+}≽\text{D}x\\ & {\text{d}}_{+}≽0 \end{array}$$

where *d*_+_ is an *N* × *M* matrix (*N* number of measurements, *M* number of sensors), *y*_t_ is the vector of *M* sensor responses at measurement *t* (*y*_t_ ∈ ℝ^M^, *t* = 1, …, *N*) and *x*_t_ is the vector of the estimated trend for each sensor at measurement *t* (x_t_ ∈ ℝ^M^, *t* = 1, …, *N*). The formula involves the computation of second order differences, hence the summations in second term of objective function starts from *t* = 3. In this case the regularization term contains *l*_p_-norms, where *p*≥ 1. The choice of the l_p_-norm induces a different way to aggregate the response of different sensors. In particular, in this work we select three different *l*_p_-norms:

*l*_1_**-norm** This norm forms the foundation of the well-known LASSO regression [33]. As already pointed out previously, the *l*_1_-norm induces sparsity, which in this case means basing the change point decision on the least number of sensors. It is worth noticing that with this norm the trend components of the individual sensors can be estimated separately.
*l*_2_**-norm** This norm is connected to the Group LASSO regression [34]. Compared with the *l*_1_-norm aggregation, this formulation couples the regularization residuals obtained for each sensor at the same time index. Therefore the detected trend shows simultaneous changes in all the sensors at common kink points.
*l*_∞_**-norm** This norm corresponds to taking the maximum of the regularization residuals obtained for each sensor at the same time index. It can be seen as an extreme case of the integration with l2-norm, since it means that all the sensors must detect a change for a certain time index.

Finally it is important to notice that the summations over time indices are equivalent to the calculation of an *l*_1_-norm along the time dimension (the arguments of the sum are norms, and hence positive), preserving the sparsity of the kinks.

## 5. Results and Discussion

Before the TREFEX algorithm can be applied, the time constants (*τ*_+_ and *τ*_−_) for each sensor need to be estimated. For this purpose, a series of 6 Step experiments (see Figure 3 (a)) per sensor were conducted. Three experiments were using ethanol as target compound while the other three used 2-propanol. The parameters were calculated by fitting an exponential to the observed transients. Since no significant variation of the time constants with respect to the concentration was observed, the mean of all the experiments is considered as a reliable estimator for the time constant of the sensors. For reference, the obtained values are shown in Table 1. These values were used in the experiments presented in the remainder of this paper.

Due to the differences in the sensing surface, different models of MOX sensors exhibit a different dynamic range. This means that some of the sensors, when responding, change their resistance value only a few Ohms while others vary hundreds or even thousands of Ohms. Thus, before running change point detection algorithms, we normalize the dynamic ranges of the sensor responses to the interval [0,1] using linear scaling.

**Figure 6.**
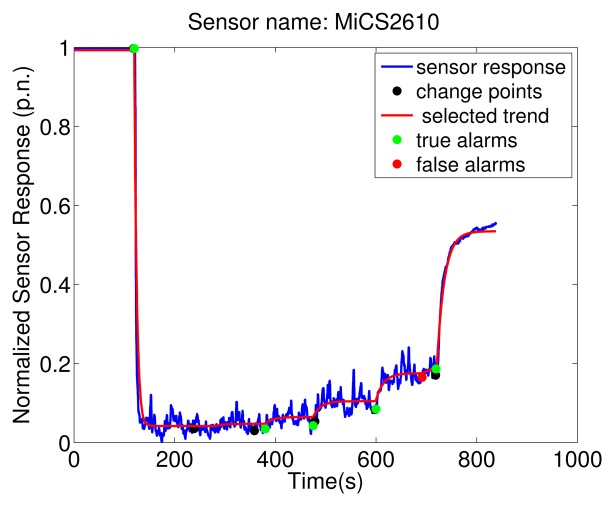
Result of the execution of the proposed algorithm for an experiment where the gas source was changing the emitted concentration using the Descending Stairway strategy (0%, 100%, 80%, 60%, 40%, 20% and 0% of gas source strength). The regularization parameter is set to *λ* = 4.

To exemplify the proposed algorithm, Figure 6 shows the MiCS 2610 sensor response and the result of TREFEX. In the depicted experiment ethanol was emitted according to the Descending Stairway pattern (see Figure 3 (c)). The six change points are marked in the figure with a black dot. Five of them were correctly identified by the algorithm. An alarm is considered a true alarm (indicated in the figure by a green dot) when it is the closest alarm to a change point. All the other alarms are considered false alarms (indicated by red dots in the figure). Besides these five correctly identified change points, two errors are observed. On one hand, the change from 100% to 80% emission rate (time 238 s) is missed by the algorithm. A smaller value of *λ* would allow to detect this change point as well. On the other hand, a false alarm is raised at time 691 s (marked by the red dot). This error could be avoided by setting a larger *λ*. This is an example of the trade-off that needs to be tackled for selecting an appropriate value of the regularization parameter *λ*.

In the following sections, we perform a numerical evaluation of the TREFEX algorithm and compare it with the GLR method presented in [35] and summarized in Section 5.2. Single sensor (Section 5.3) and sensor array (Section 5.5) configurations are considered. The strategy for selecting the value of the regularization parameter *λ* is evaluated in Section 5.4.

### 5.1. Evaluation Methodology

The proposed change detection method returns a list of change points, which we will call alarms. Additionally, for our experiments the ground truth dataset that contains the time of the actual change points is available. *A true alarm* is defined as the closest alarm to a change point. An alarm that is not a true alarm is a *false alarm*. According to these definitions, TP (true positive) is given by the number of true alarms. FP (false positive) is given by the number of false alarms and FN (false negatives) is given by the number of change points minus the number of true alarms. From these numbers we compute the well-known performance metrics precision and recall:

(10) $$\text{Precision}=\frac{TP}{TP+FP}\;\text{Recall}=\frac{TP}{TP+FN}$$

A maximum precision value is achieved when all the alarms are true change points. A maximum recall value means that no true change point was missed by the algorithm. The perfect algorithm maximizes both values, *i.e.*, detects all change points without raising false alarms.

A value of the regularization parameter *λ* corresponds the trade-off between precision and recall. A common method to formalize this trade-off is the harmonic mean of precision and recall, known as *F*-*measure*:

(11) $$F=2\times \frac{\text{precision}\times \text{recall}}{\text{precision}+\text{recall}}$$

The *F*-*measure* can be used to compare points on the *precision*-*recall curve*, obtained for different values of *λ*. The *F*-*measure* is used as evaluation criterion in this paper; higher values correspond to a better performance.

Another evaluation criterion is based on how close (in time) the true alarms are to the change points. To this respect we define the Average Distance (AD) performance measure as sum of the distance between the true alarms and the corresponding change points divided by the number of true alarms.

(12) $$AD=\frac{\sum_{i=1}^{TP}\;\left|t_{i}^{ta}-t_{i}^{cp}\;\right|}{TP}$$

$t_{i}^{cp}$ indicates the time the *i*-th change point happened and $t_{i}^{ta}$ is the associated true alarm produced by the algorithm.

### 5.2. Generalized Likelihood Ratio

The Generalized Likelihood Ratio (GLR) approach is summarized here since it is compared with the TREFEX algorithm below. We compare against GLR, because, to our best knowledge, it is the only change point detection algorithm that was specifically applied to MOX sensors.

GLR calculates the likelihood ratio between the hypotheses of having a change point at sample *j versus* the hypothesis of not having a change point:

(13) $$g_{k}=\underset{1\;\leq j\;\leq k}{\max}\underset{\theta_{1}}{\sup}\left(\sum_{i=j}^{k}\ln\frac{p_{\theta_{1}}\;(x_{i}\;)}{p_{\theta_{0}}\;(x_{i}\;)}\;\right)$$

The likelihoods are based on a parametric probability distribution function, which is governed by a set of parameters *θ*. In this case we consider Gaussian distributions so *θ* is represented by mean and variance. *θ*_0_ denotes the mean/variance estimated using all samples in the time interval. *θ*_1_ denotes the mean/variance estimated using only the samples collected after a hypothetical change point *j*. If *g*_k_ is above a preselected threshold *h*, then a change point is declared and the data collected before the change point are not considered any longer to detect new change points.

### 5.3. Single Sensor Analysis

**Figure 7.**
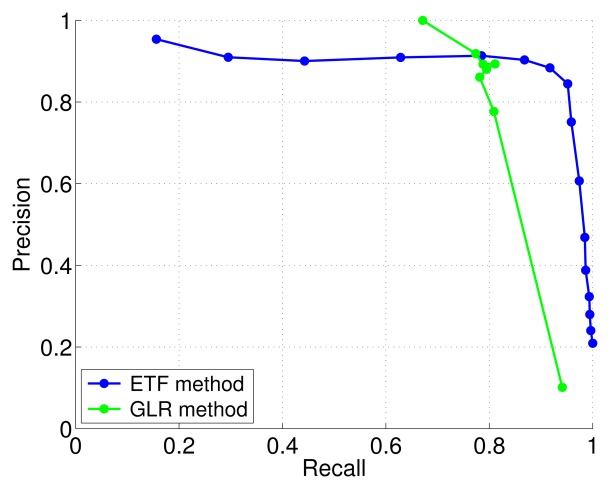
*Precision-recall curve* for TREFEX and GLR algorithms. The considered sensor is the MiCS 2610.

In Figure 7 an example of precision-recall curve comparing TREFEX and GLR for the MiCS 2610 sensor averaged over 54 experiments is shown. Qualitatively similar graphs were obtained for the other sensors. The maximum *F-measure* is attained at the point of the *precision-recall curve* that is closest to the upper right corner. It is clear from the figure that the maximum F-measure attained by the TREFEX algorithm is higher than the one obtained by GLR. Table 2 shows how TREFEX outperforms GLR for each of the considered sensors.

Additionally, the ranking of the sensors with respect to the *F-measure* provided in Table 2 shows that both approaches achieve the best results with the same three sensors (MiCS 2710, MiCS 5135 and MiCS 2610).

Moreover, Table 2 shows the average distance (AD) between true alarms and change points across all the 54 experiments. Also with respect to this performance measure, TREFEX clearly outperforms GLR since true alarms are in all cases much closer (only half the deviation) to the real change points. The principle behind the GLR algorithm is to accumulate enough evidence of changes before declaring an alarm, but the first steps after a change look no different than the old signal plus noise. Hence the change is detected somewhere on the slope of the exponential sensor response and this introduces some offset. In contrast, by fitting exponentials to the signal, TREFEX is able to pinpoint the time of the change more accurately.

### 5.4. Parameter Selection

**Figure 8.**
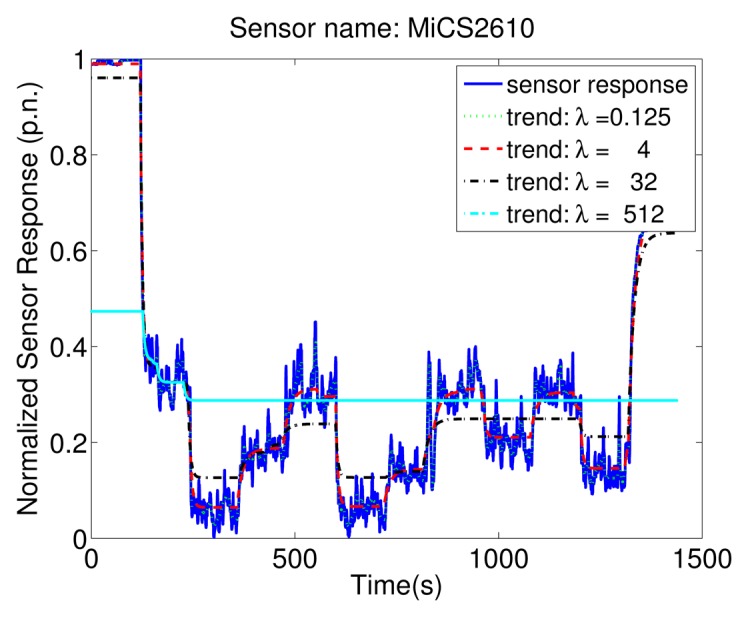
Estimated trends for the response of the MiCS 2610 for an experiment where the gas source was changing the emitted concentration. Corresponding regularization parameters for the illustrated trends are *λ* = 0.125, *λ* = 4, *λ* = 32 *and λ* = 512.

Figure 8 shows how different values of the regularization parameter *λ* control the trade-off between preserving the signal in the estimated trend and representing it by a small number of exponential functions. In case of *λ* = 0.125 the estimated trend is very similar to the signal, making the identification of real change points hard. For *λ* = 512 the estimated trend misses many of the change points. With the parameter selection method discussed in Section 4.3 the selected *λ* for this experiment is 4. This value results in a trend that does not miss any of the change points and does not raise any false alarms.

Directly selecting the *λ* based on the *F-measure* is not possible for most applications, since it requires to know the change points' ground truth. We show that our substitute of minimizing the distance *δ* in the trade-off curve (see Section 4.3) is highly correlated to maximizing the *F-measure*. This means that points close to the origin in the trade-off curve (see Figure 5) correspond to a high *F-measure*. Using the available data, the distance *δ* and the *F-measure* were computed for 13 different values of *λ* (*λ* = [2^−4^, 2^−3^,…, 2^8^]). As can be seen in Table 3, the linear correlation analysis between both values was performed using the Pearson coefficient and showed a strong negative correlation. Hence, we conclude that the distance to the origin is a suitable heuristic to select the hyper-parameter *λ*.

### 5.5. Sensor Array Analysis

In general, the combination of multiple sensors aims at increasing the robustness and reliability of the overall change point detection. Here we present numerical results on how the different norms proposed in Section 4.4 perform for aggregating the response of sensors deployed in a sensor array. Table 4 reports the performance based on the maximum *F-measure* obtained for the sensor array (all 11 sensors) with *l*_1_-norm, *l*_2_-norm and *l*_∞_-norm.

The best combination result for the sensor array was achieved using *l*_2_-norm. The sensor array aggregation using the l2-norm achieves a higher *F-measure* than eight of the eleven sensors and slightly worse than the three best performing sensors (MiCS 2610, MiCS 2710, and MiCS 5135, see Table 2). The results for the sensor array are slightly worse than the best results obtained with a single sensor. A likely reason is that the p-norm couples the individually detected change points in a specific way that may either discard correct individual change point detections or consider too many erroneous change detections. Low values for the average difference between detected and ground truth change point time are achieved for all considered norms.

**Figure 9.**
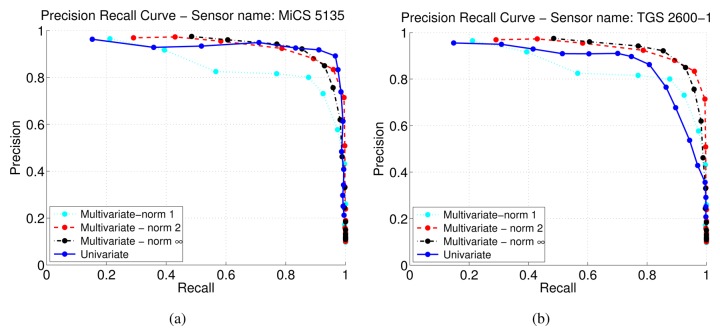
Comparison of *precision-recall curves* of a single sensor and the sensor array with different norms using the exponential trend filtering method.

In Figure 9, we compare the sensor array results to single sensor performances. Figure 9(a) presents the case in which the single sensor (MiCS 5135) has a higher maximum *F-measure* than the sensor array results. Figure 9(b) shows the comparison with another single sensor (TGS 2600-1). In this case the results obtained aggregating the sensors with *l*_2_-norm and *l*_∞_-norm clearly outperform the single sensor.

Combining the sensors using *l*_1_-norm, which corresponds to the sum of the individually calculated kink vectors, does not perform well in general, because it aggregates all the false alarms. This leads to lower precision values, as shown in Figure 9.

The evaluation of the parameter selection for the sensor array case has been done in a similar way for the single sensor parameter selection. Table 5 shows the Pearson correlation between the Euclidean distances to the origin of the trade-off curve and the *F-measure* for different norms. The Euclidean distance *δ* in the trade-off curve and the *F-measure* were computed for 13 different values of *λ* (*λ* = [2^−4^, 2^−3^, …, 2^8^]). Again, the Pearson correlation coefficients show highly negative values, confirming the usefulness of the heuristic for the sensor array as well.

Table 6 reports the performance of TREFEX and GLR for some selected subsets of the sensor array. The selected subsets are the same as in [35]. Again, for the sensor array configuration the TREFEX algorithm outperforms GLR both with respect to the maximum *F-measure* and with respect to the average distance of the alarm from the change points.

## 6. Conclusions and Future Work

In this paper we introduced TREFEX, a novel change point detection algorithm, especially designed for MOX gas sensors in an open sampling system. TREFEX models the response of a MOX sensor as a piecewise exponential signal, and considers the junctions between consecutive exponentials as change points. TREFEX can be used for single MOX sensors or for arrays of MOX sensors. Moreover, since TREFEX is designed to detect exponential trends, it can be applied to any kind of first order sensor. Along with the proposed algorithm, a parameter selection method for choosing the regularization parameter is suggested. The performance of the algorithm is evaluated experimentally for different sensors and scenarios and compared with the previously proposed GLR method.

Our results show that solving the change detection problem for MOX gas sensors with the TREFEX algorithm is advantageous compared with the GLR method in several ways.

First, and most importantly, TREFEX outperforms the GLR method. The maximum F-measure is higher for all considered sensors and experiments. Second, the algorithm does not involve a tuning parameter that has to be set arbitrarily by the user. Instead, an automatic parameter selection step is provided with the algorithm. Third, the computational requirements for TREFEX are lower. While GLR scales quadratically with the length of the considered signal, TREFEX scales linearly. Last but not least, the estimate that TREFEX provides about the position of the change points in time is much more accurate. GLR requires accumulating enough evidence before it declares a change, while TREFEX, due to considering the whole signal, is able to detect the position of the change points more accurately.

One of the major improvement to the presented TREFEX algorithm will be its extension to an on-line version based on the work in the area of continuous-time trend filtering [18]. Another important step is its application to scenarios in uncontrolled outdoor environments. While for developing algorithms carefully controlled experiments are necessary, relevant applications for open sampling systems are far more challenging. Hence the next step for us is to apply the algorithm to sensors and sensor arrays mounted on a mobile robot platform and use the detected changes in the gas concentration to build maps with them [5].

## Figures and Tables

**Table 1.** Time constants calculated of the rise and decay phase of each sensor. The sensors were used without any modifications to improve response and decay characteristics.

**Sensor**	**T.C. Response (s)**	**T.C. Decay (s)**
MiCS 2610	4.96	14.92
MiCS 2710	17.18	23.69
MiCS 5521(1)	2.43	5.54
MiCS 5121	5.97	10.72
MiCS 5135	5.37	14.65
MiCS 5521(2)	3.13	5.20
TGS 2600(1)	5.22	19.31
TGS 2611	3.52	7.36
TGS 2620	3.29	15.58
TGS 2600(2)	5.19	19.74
TGS 2602	4.84	36.25

**Table 2.** Comparison between the TREFEX and the GLR algorithm. TREFEX outperforms GLR both in terms of the maximum *F-measure* and the average distance of the alarms from the change points. The value of the selected parameters (*λ* for TREFEX, *h* for GLR) are also given. The ranking of the sensors w.r.t. maximum *F-measure* shows good agreement between the two algorithms regarding which of the sensors are most suitable to detect change points for the given problem.

**Model**	**Exponential Trend Filtering**				**GLR Method**			

	**Rank**	**Maximum F-measure**	*λ*	**Average Distance(s)**	**Rank**	**Maximum F-Measure**	*h*	**Average Distance(s)**
MiCS 2610	**3**	**0.90**	8	3.72	**3**	**0.85**	90	7.85
MiCS 2710	**1**	**0.95**	16	4.78	**2**	**0.87**	120	11.12
MiCS 5521(1)	11	0.77	8	5.59	10	0.70	88	11.52
MiCS 5121	4	0.88	16	5.19	4	0.82	91	9.82
MiCS 5135	**2**	**0.92**	8	4.17	**1**	0.87	93	8.60
MiCS 5521(2)	8	0.81	16	5.05	6	0.77	82	11.61
TGS 2600(1)	6	0.83	4	6.05	7	0.72	107	11.14
TGS 2611	5	0.84	16	5.51	5	0.79	93	11.52
TGS 2620	9	0.80	4	5.89	8	0.72	93	12.26
TGS 2600(2)	7	0.83	4	6.40	9	0.72	93	11.63
TGS 2602	10	0.78	0	6.57	11	0.56	65	13.42

**Table 3.** Pearson correlation coefficient between the Euclidean distance *δ* of the trade-off curve to the origin and *F-measure* for each sensor. The strong correlation shows that the distance to the origin is a suitable heuristic to select the hyper-parameter *λ*.

**Sensor**	**MiCS**						**TGS**					

	**2610**	**2710**	**5521(1)**	**5121**	**5135**	**5521(2)**	**2600(1)**	**2611**	**2620**	**2600(2)**	**2602**
**Correlation**	−0.88	−0.81	−0.69	−0.79	−0.81	−0.50	−0.84	−0.73	−0.88	−0.86	−0.73

**Table 4.** Comparison of different norms used to combine the answers of the sensor array.

**Model**	**Exponential Trend Filtering**			

	**Rank**	**Maximum F-Measure**	*λ*	**Average Distance(s)**
Multivariate - *l*_1_-norm	3	0.83	32	4.20
Multivariate - *l*_2_-norm	1	0.89	32	3.98
Multivariate -*l*_∞_-norm	2	0.88	64	3.92

**Table 5.** Pearson correlation between Euclidean distances *δ* to the origin of the trade-off curve and the *F-measure* for different norms.

**Model**	**Multivariate**		

	*l*_1_-**norm**	*l*_2_-**norm**	*l*_∞_-**norm**
**Correlation**	−0.72	−0.67	−0.66

**Table 6.** Maximum *F-measure* calculated by Multivariate TREFEX using *l*_2_-norm and Multivariate GLR for selected subset of the array. The corresponding hyper-parameters and detection intervals of maximum *F-measure* are also presented.

**Model**	**Exponential Trend Filtering**			**GLR Method**		

	**Maximum F-Measure**	*λ*	**Average Distance(s)**	**Maximum F-Measure**	*h*	**Average Distance(s)**
MiCS 2710-MiCS 5521(2)	0.91	16	4.23	0.87	232	9.13
MiCS 2710-TGS 2602	0.92	16	4.37	0.84	205	12.41
MiCS 2710-TGS 2602-MiCS 5521(2)	0.90	16	4.11	0.86	209	8.68
MiCS 5521(2)-TGS 2602-MiCS 2610	0.86	32	3.43	0.84	182	8.08
MiCS 2610-TGS 2611-TGS 2602	0.88	16	3.81	0.85	220	8.16
